# Multicolor two-photon imaging of endogenous fluorophores in living tissues by wavelength mixing

**DOI:** 10.1038/s41598-017-03359-8

**Published:** 2017-06-19

**Authors:** Chiara Stringari, Lamiae Abdeladim, Guy Malkinson, Pierre Mahou, Xavier Solinas, Isabelle Lamarre, Sébastien Brizion, Jean-Baptiste Galey, Willy Supatto, Renaud Legouis, Ana-Maria Pena, Emmanuel Beaurepaire

**Affiliations:** 1 Laboratory for Optics and Biosciences, Ecole polytechnique, CNRS, INSERM, Université Paris-Saclay, 91128 Palaiseau cedex, France; 2 L’Oréal Research and Innovation, 93600 Aulnay sous Bois, France; 3 Institute for Integrative Biology of the Cell (I2BC), CEA, CNRS, Univ. Paris-Sud, Université Paris-Saclay, 91198 Gif-sur-Yvette, France

## Abstract

Two-photon imaging of endogenous fluorescence can provide physiological and metabolic information from intact tissues. However, simultaneous imaging of multiple intrinsic fluorophores, such as nicotinamide adenine dinucleotide(phosphate) (NAD(P)H), flavin adenine dinucleotide (FAD) and retinoids in living systems is generally hampered by sequential multi-wavelength excitation resulting in motion artifacts. Here, we report on efficient and simultaneous multicolor two-photon excitation of endogenous fluorophores with absorption spectra spanning the 750–1040 nm range, using wavelength mixing. By using two synchronized pulse trains at 760 and 1041 nm, an additional equivalent two-photon excitation wavelength at 879 nm is generated, and achieves simultaneous excitation of blue, green and red intrinsic fluorophores. This method permits an efficient simultaneous imaging of the metabolic coenzymes NADH and FAD to be implemented with perfect image co-registration, overcoming the difficulties associated with differences in absorption spectra and disparity in concentration. We demonstrate ratiometric redox imaging free of motion artifacts and simultaneous two-photon fluorescence lifetime imaging (FLIM) of NADH and FAD in living tissues. The lifetime gradients of NADH and FAD associated with different cellular metabolic and differentiation states in reconstructed human skin and in the germline of live *C. Elegans* are thus simultaneously measured. Finally, we present multicolor imaging of endogenous fluorophores and second harmonic generation (SHG) signals during the early stages of Zebrafish embryo development, evidencing fluorescence spectral changes associated with development.

## Introduction

Multiphoton microscopy is a powerful tool for label-free and non-invasive functional imaging in small organisms and tissues^1, 2^. Pulsed near infrared excitation light allows in-depth imaging based on contrasts such as endogenous fluorescence^2^, second harmonic generation (SHG)^3^ and third harmonic generation (THG)^4^. Endogenous fluorescence in living tissues arises from several intrinsic biomarkers that play important roles in physiological processes^2^. The primary intracellular sources are NAD(P)H and FAD, the two major cofactors of redox reactions in the cell and central regulators of energy production and metabolism^5, 6^. Their fluorescence reports on the metabolic activity of cells allowing tissue physiology and processes such as stem cell differentiation, cancer development and neurodegenerative diseases to being non-invasively monitored^7–12^. The fluorescence lifetimes of NADH and FAD are different upon binding to the protein during the electron transport chain. FLIM provides sensitive measurements of the free and protein-bound NAD(P)H ratio and of the redox states (NADH/NAD^+^) of cells, and can be used to distinguish glycolytic and oxidative phosphorylation metabolic states^13–17^. Monitoring lifetime of free and protein-bound FAD has also been exploited to quantify redox ratio FAD/FADH_2_, and used as a biomarker of precancerous epithelial cells^12^. It is well established that retinoids play a crucial role in stem cell differentiation and embryo development^18, 19^ and their concentration and gradients have been detected *in vivo* during zebrafish development^9, 20^. Other intrinsic fluorophores such as porphyrin, collagen, elastin, keratin, lipofuscin and melanins have relevant roles in several physiological processes and diseases such as cancer and multiphoton microscopy can provide functional imaging in a non-invasive way^21–24^.

While several advanced approaches have been developed to improve identification and quantification of endogenous fluorophores based on fluorescence lifetime imaging^9^, spectrally resolved detection^23, 25^, and spectrally resolved FLIM^26–28^, multiphoton microscopy of endogenous fluorophores and its applications to live imaging are still limited by acquisition speed and challenging multicolor excitation. Being able to efficiently image multiple endogenous fluorophores simultaneously in tissues is a paramount objective, as it could possibly allow physiological and pathological processes to being tracked in dynamic systems. Endogenous fluorophores have non-overlapping two-photon absorption maxima in the range between 700 nm and 1000 nm^2^ and are present in a wide variety of concentrations in living tissues. Imaging of multiple endogenous fluorophores is usually performed by sequential excitation at different wavelengths using a tunable laser, leading to imaging rates decreasing with the number of excitation wavelengths, and difficulties in ensuring pixel-level registration between the channels in the case of a dynamic sample. For example, NADH and FAD are often sequentially excited at 750 nm and 880 nm ^12, 22, 29–31^. Strategies conceived so far to simultaneously excite NADH and FAD with one single wavelength^32^ can compensate the difference in absorption spectra and concentrations with the payoff of low excitation efficiency.

To overcome these limitations, a mixed-wavelength excitation^33^ was implemented to achieve multicolor two-photon imaging of endogenous fluorophores in living tissues. Wavelength mixing was recently demonstrated to being an effective approach for achieving simultaneous two-photon excitation of three fluorescent proteins with non-overlapping spectra, while being compatible with both standard point-scanning^33^ and light sheet^34^ excitation geometries. A distinct advantage of wavelength mixing for ratiometric measurements in the point-scanning geometry is that the fluorescence images are intrinsically co-registered at the scale of the diffraction-limited excitation volume^33^. We report here on the implementation of this strategy to the excitation of spectrally distinct endogenous fluorophores and optical redox ratio imaging, overcoming the difficulties associated with sequential excitation at different wavelengths such as motion artifacts in living samples. We also demonstrate multicolor two-photon FLIM to characterize the microenvironment of endogenous fluorophores and their binding properties. Through a simultaneous excitation of NADH and FAD in living tissues, reliable 2-photon ratiometric redox imaging and simultaneous FLIM of NADH and FAD are achieved with similar signal levels in both channels. Measurements of NADH and FAD lifetime gradients associated with cellular differentiation in living tissues such as reconstructed human skin and *C. Elegans* germ line are also presented.

Finally, a simultaneous imaging of several endogenous fluorophores and SHG signals in living zebrafish embryos is demonstrated.

## Results

### Efficient multicolor two-photon fluorescence lifetime imaging of endogenous fluorophores in living tissues

**Figure 1:**
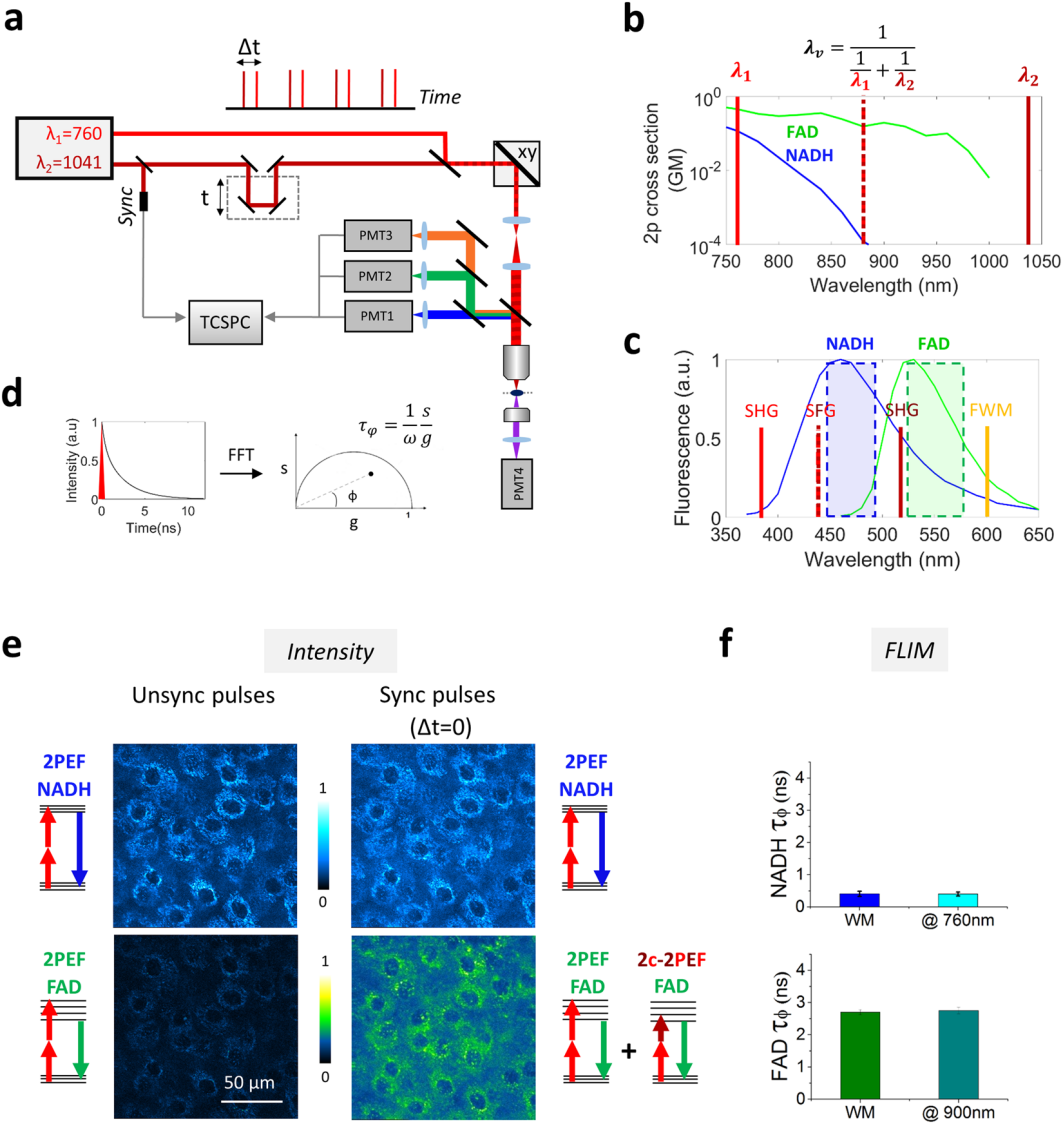
Principle of efficient multicolor two-photon fluorescence lifetime imaging of endogenous fluorophores (**a**) Pulse trains from dual-output femtosecond laser (λ1 = 760 nm, λ2 = 1041 nm) are synchronized using a delay line and co-aligned in the microscope. Pulse synchronization gives rise to two-beam processes such as sum-frequency generation (SFG), two-color two-photon excited fluorescence and four wave mixing (FWM). Fluorescence signals are epi-detected in three different spectral channels, while SHG is forward detected. Time-correlated single photon counting (TCSPC) electronics measures the arrival time of the fluorescence photons with respect to the laser pulse. (**b**) Two-photon cross sections of NADH and FAD. By synchronizing the two beams we create a virtual wavelength for two-photon excitation λ_v_ = 2/(1/λ_1_ + 1/λ_2_) that corresponds to 879 nm. (**c**) Emission filters are chosen to select the emission of NADH and FAD and reject coherent signals such as SHG, SFG and FWM. (**d)** The multi-exponential fluorescence intensity decay is transformed with a Fourier transform (FFT) and the real (g) and imaginary (s) parts are plotted in the graphical phasor plot. (**e**) Two channels fluorescence images of NADH and FAD in reconstructed human skin using synchronized (Δt = 0) and unsynchronized (Δt = 1 ps) beams. Two-photon-excited fluorescence (2PEF) for NADH and FAD fluorophores occurs when fluorophores are excited at λ_1_. When the beams are synchronized (Δt = 0), FAD fluorescence is enhanced by two-color two-photon excited fluorescence (2c-2PEF). (**f**) Fluorescence lifetime of NADH and FAD is not affected when the fluorophores are two-photon excited by single wavelength or wavelength mixing. Measurements were performed in solution.

The principle of efficient multicolor two-photon FLIM of endogenous fluorophores is described in Fig. 1. To obtain simultaneous multicolor two-photon excitation by wavelength mixing over the 760–1040 nm range, we spatially and temporally overlapped two synchronous pulse trains produced by a commercial dual-output femtosecond laser at λ_2_ = 1041 nm (fixed output) and λ_1_ = 760 nm (tunable output) (Fig. 1a and Methods). In this configuration, the two beams separately generate one-color nonlinear processes, namely two-photon excited fluorescence (2PEF) of blue and red chromophores and second harmonic generation (SHG) (Fig. 1b and c). The spatiotemporal overlap of the two pulse trains provides an additional excitation route (Fig. 1b) via two-color two-photon excited fluorescence (2c-2PEF)^33^, which is equivalent to having a virtual third laser providing two-photon excitation at λ_v_ = 2/(1/λ_1_ + 1/λ_2_) = 879 nm, *i.e*. well suited for independent excitation of FAD. Of note, additional nonlinear processes can result from dual-beam excitation, such as sum frequency generation (SFG) and four wave mixing (FWM) (Fig. 1b and c). Fluorescence signals were epi-detected (Fig. 1a) in three different spectral ranges (blue, green and red), while SHG signal was forward detected (Fig. 1a). Appropriate emission filters (Fig. 1c) were chosen to reject coherent signals (SHG, SFG, FWM) from the fluorescence channels.

FLIM was implemented with a custom made time-correlated single photon counting (TCSPC) system providing simultaneous channels with 24 temporal bins of 500 ps (see Methods). The FLIM images were analyzed through phasor analysis^9, 35^ (Fig. 1d) and represented with the *τ*_*φ*_ contrast (see Methods). Two-color two-photon excitation increased FAD excitation efficiency (Fig. 1e and S1d) compared to excitation at 760 nm only (Fig. 1e), sequential single wavelength excitation at 760 nm and 900 nm respectively (Figs S1b and d) and single wavelength excitation at 800 nm (Fig. S1c and d), resulting in higher SNR and accuracy in the FLIM measurement (Figs S1d, S2c and e). As shown on Fig. 1f, the measured fluorescence lifetime values of NADH and FAD were not affected by the wavelength mixing acquisition strategy.

### Simultaneous excitation of NADH and FAD by wavelength mixing for fast redox imaging and FLIM reveals a metabolic gradient associated with cellular differentiation in living tissues

Mixed wavelength two-photon excitation provides independent control of the excitation efficiency in three separate spectral ranges^33^, through the adjustment of the laser powers and of the delay between the pulse trains. We optimized the 2-photon excitation efficiency of NADH and FAD by adjusting the power of the two laser beams (Fig. 1a), resulting in simultaneous images of comparable intensity despite the disparity in fluorophore concentrations (Fig. 1e, Methods).

Emission filters were chosen to minimize fluorescence bleed-through between the two channels. Figure S3 shows cells imaged using this approach. The molecular origin of the fluorescence detected in each channel was checked by submitting cells to the oxidative phosphorylation inhibitor potassium cyanide (KCN) that prevented the oxidation of NADH. This treatment resulted in an increase in the NADH fluorescence intensity (84 ± 30%) and a concurrent reduction in the FAD intensity signal (15 ± 9%) (Fig. S3a and c), confirming that the predominant sources of signal were NADH fluorescence in the “blue” channel and FAD in the “green” channel. The metabolic drug had different impacts upon the fluorescence lifetimes of the two metabolites (Fig. S3b and d). Upon application of KCN, the measured NADH fluorescence lifetime significantly decreased from (1.87 ± 0.09) ns to (1.14 ± 0.1) ns, while the FAD lifetime only slightly decreased from (1.31 ± 0.04) ns to (1.10 ± 0.06) ns.

**Figure 2:**
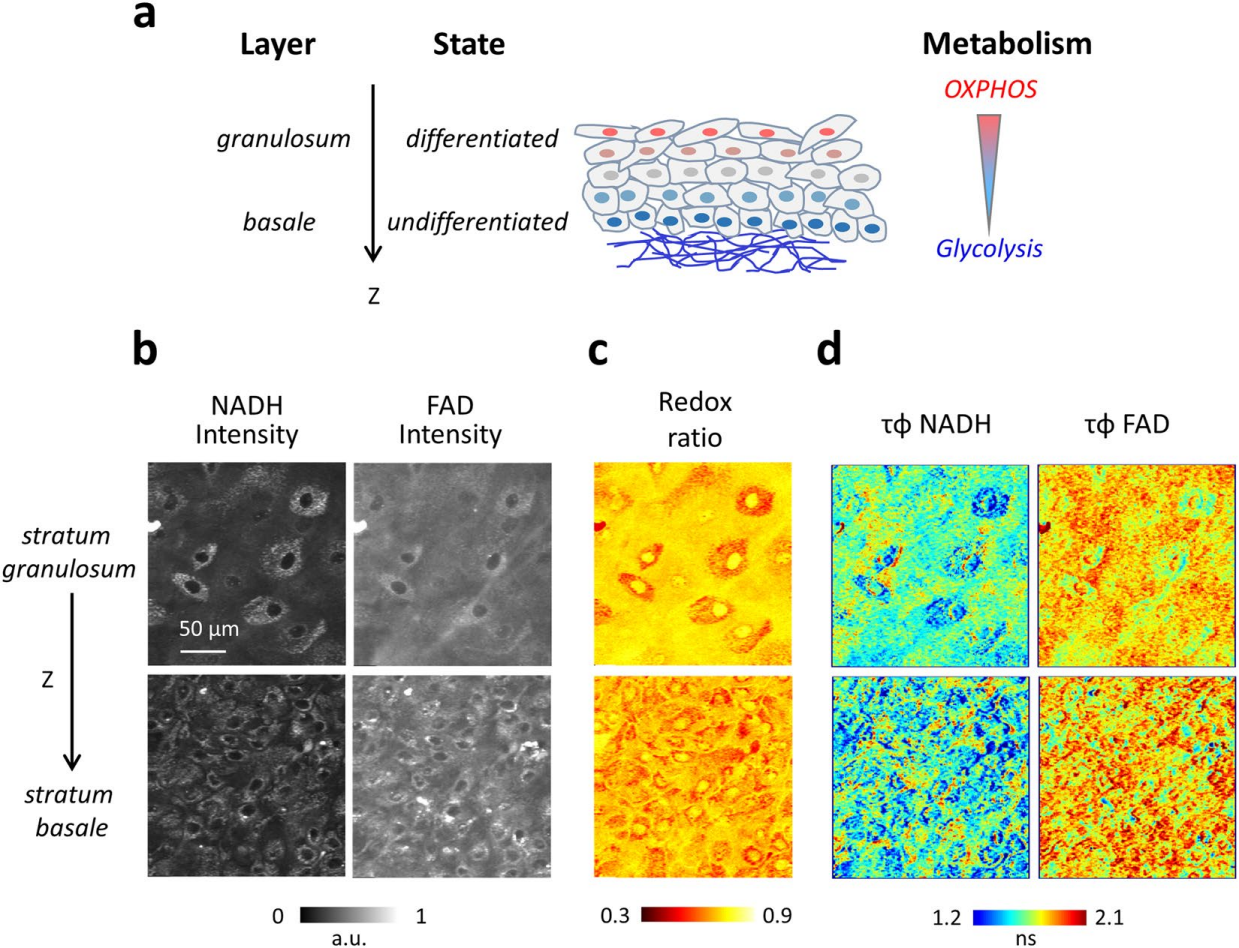
Simultaneous multiphoton FLIM imaging of NADH and FAD reveals a metabolic gradient in redox ratio and lifetime in reconstructed human skin associated with cell differentiation (**a**) Three-dimensional scheme of reconstructed human skin. Proliferating cells are located at the basal layer, right above the dermis, while differentiated cells are located on different layers above the basal layer. A metabolic gradient along the z-depth of the epidermis goes from an energetic metabolism dominated by glycolysis in the basal cells to one dominated by OXPHOS in the differentiated cells. (**b**) NADH and FAD intensities are acquired simultaneously at different depths of the epidermis. Two representative images are shown at two different depths within *stratum basale* and *stratum granulosum*. (**c**) Intensity redox ratio FAD/(NADH + FAD), calculated simultaneously from NADH and FAD intensities reveals heterogeneity within single planes and a z-gradient associated with cell differentiation. (**d**) NADH and FAD lifetime along the z-depth reveals a gradient associated with cell differentiation. Time per pixel, 160 µs. Triplicate experiments were performed.

**Figure 3:**
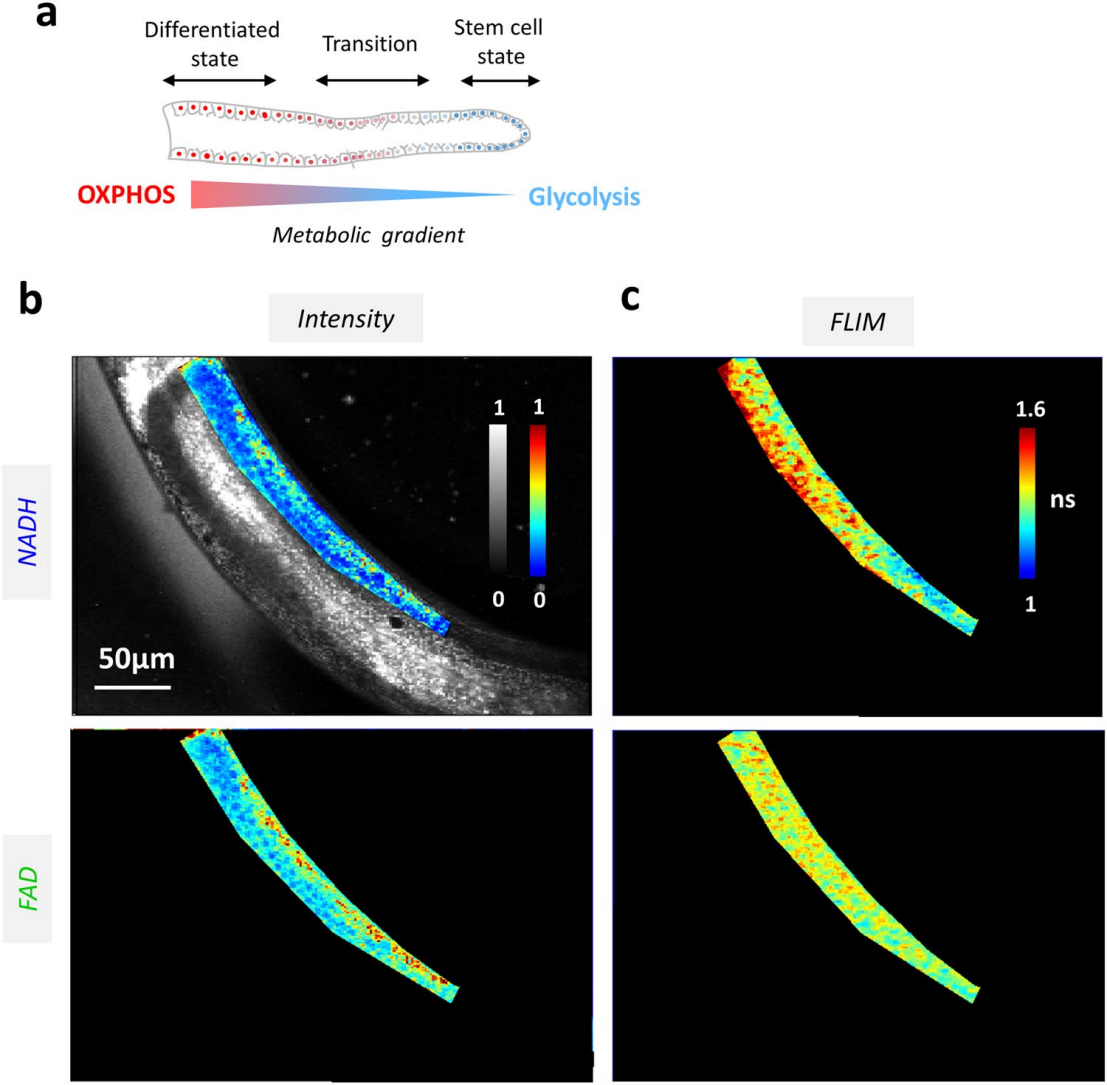
Simultaneous multiphoton FLIM imaging of NADH and FAD reveals a metabolic gradient associated with stem cell differentiation in *C.Elegans* germ line (**a**) Scheme of the germ line of the *C. Elegans*. Stem cells that are located at the tip of the germ line; a metabolic gradient along the axis of the germ line goes from an energetic metabolism dominated by glycolysis in proliferating stem cells to one dominated by OXPHOS in the differentiated cells^39^. (**b**, **c**) NADH and FAD intensities (**b**) and lifetimes (**c**) are simultaneously acquired. Time per pixel, 160 µs. Triplicate experiments were performed.

The metabolic imaging using NADH and FAD signals was performed in two tissue models for cell differentiation, namely reconstructed human skin (Fig. 2) and *C. Elegans* germ line (Fig. 3). Most proliferative cells rely on aerobic glycolysis in contrast to normal differentiated cells which rely primarily on oxidative phosphorylation (OXPHOS)^36, 37^. During proliferation, the large increase in glycolytic flux rapidly generates cytosolic ATP resulting in high NADH/NAD+ and free/bound NADH ratios^15, 36, 38^ (Fig. S4). Conversely, cells that are characterized by an OXPHOS phenotype usually present low NADH/NAD+ and low free/bound NADH ratios.

The simultaneous NADH and FAD signals with comparable intensities were at first measured in reconstructed human epidermis (see Methods) (Fig. 2a and b), achieving an intensity redox imaging in a one-shot measurement (Fig. 2c and S5c). Reconstructed human skin is constituted by a fibroblasts-embedded collagenous substrate supporting successive layers of keratinocytes: the basal layer, located right above the substrate, contains proliferating undifferentiated cells in a hypoxic environment, while the upper layer *stratum granulosum* contains differentiated keratinocytes in a normoxic environment. A gradient in cell differentiation state is present between these two layers as a function of depth, which is associated with a metabolic gradient with different balance between Glycolysis and OXPHOS mechanisms (Fig. 2a). 3D image z-stacks of reconstructed human skin samples were recorded, showing a decrease in the redox ratio (FAD/(NADH + FAD)) with depth (Fig. 2c). This indicates an increase of the redox ratio with cell differentiation and oxygen availability, in agreement with their expected metabolic phenotype^12, 31, 37^.

In addition, NADH and FAD fluorescence lifetimes were simultaneously evaluated in the same samples, showing heterogeneity in the tissue and among cells (Fig. 2d). As shown in Fig. 2d, NADH lifetime exhibits smaller values in the undifferentiated basal epithelial cells as compared to the differentiated superficial layers such as *stratum granulosum*. The smaller NADH fluorescence lifetime in the basal layer indicates an increased free/bound NADH ratio, i.e. an energetic metabolism dominated by glycolysis. In the differentiated layers, the increase in NADH lifetime is an indicator of the presence of more bound NADH, i.e. an energetic metabolism dominated by oxidative phosphorylation. These observations are consistent with previously reported results using sequential acquisition schemes^12^, and therefore validate the mixed wavelength approach for one-shot multiphoton redox imaging. We also observed an increase in FAD lifetime with the depth (Fig. 2d).

Our *in vivo* method was then applied to simultaneous NADH and FAD fluorescence lifetime measurements in a live model: the germ line of adult *C. Elegans* worms (Fig. 3). This organ exhibits a characteristic spatial pattern of cell differentiation: the tip of the germ line is made of the stem cells characterized by a glycolytic metabolic phenotype (Fig. 3a and S4b), while along the germ line there is a gradient of cell differentiation associated with a metabolic gradient from glycolysis to OXPHOS (Fig. 3a and S4a)^9, 39^.

Simultaneous NADH-FAD fluorescence lifetime images were recorded with comparable intensities (Fig. 3b and c). The data revealed a lifetime gradient in both channels along the germ line (Fig. 3c). The NADH fluorescence lifetime gradient was particularly pronounced. The tip of the germ line where the stem cells are located was characterized by a short lifetime (1.2 ± 0.1 ns), revealing an elevated ratio of free/bound NADH^9, 17^ and a glycolytic phenotype (Fig. S3b). A gradual increase from (1.2 ± 0.1) ns to (1.6 ± 0.2) ns in NADH fluorescence lifetime was found along the germ line axis, indicating a decrease in free/bound NADH ratio associated with a shift toward OXPHOS metabolic phenotype and possibly increased oxygen availability. A less pronounced FAD fluorescence lifetime gradient along the germ line was also detected (Fig. 3c).

### Multicolor two-photon imaging of endogenous signals during zebrafish embryo development

**Figure 4:**
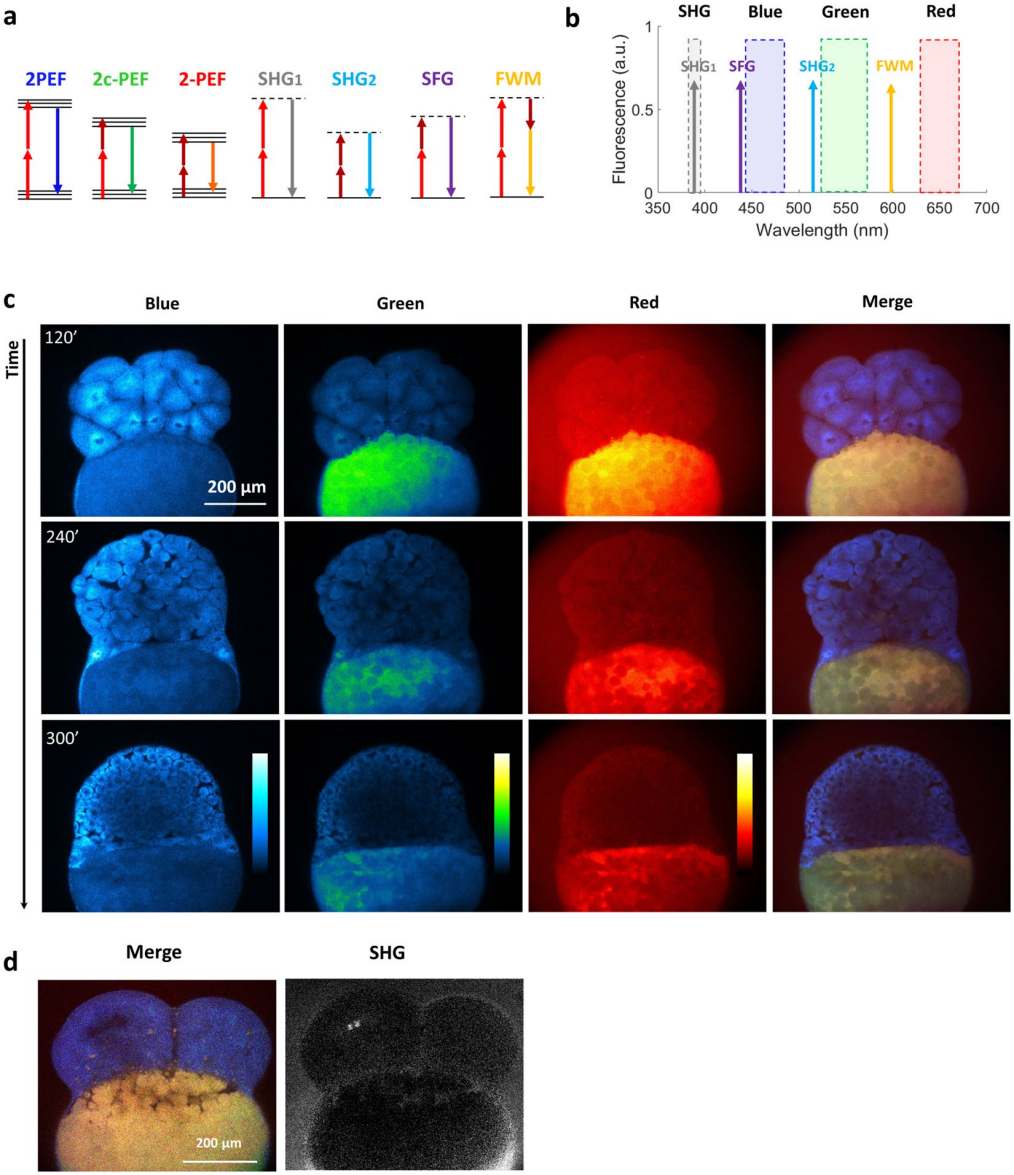
Multicolor two-photon efficient imaging of multiple endogenous fluorophores during early stages of zebrafish embryo development (**a**) Energy diagrams of two-photon excited fluorescence (2PEF) for blue and red fluorophores, two-color two-photon excited fluorescence (2c-2PEF) for green fluorophores, second harmonic generation (SHG), sum frequency generation (SFG) and four wave mixing (FWM). (**b**) Bandpass filters are chosen to select the emission of blue, green and red fluorophores rejecting coherent signals such as SHG, SFG and FWM. (**c**) Images of the blue, green, red fluorescence channel and SHG of the same zebrafish embryo at three different time points of development. Images of merged channels represent a shift in the spectroscopic characteristics of the yolk. (**d**) Multicolor two-photon and SHG images of the zebrafish embryo at 4 cell stage. Time per pixel, 40 µs. This experiment has been repeated in three independent samples.

Apart from providing a working scheme for simultaneous FAD/NADH redox ratio and fluorescence lifetime measurements, two-photon wavelength mixing in the 700–1040 nm range more generally opens the way to one-shot spectral imaging of the principal sources of endogenous fluorescence in tissues. We explored the possibility of achieving multiphoton imaging of endogenous fluorophores combined with SHG imaging in developing zebrafish embryos (Fig. 4)

As previously mentioned, the tunable laser line set to 760 nm (λ_1_ in Fig. 1a) was mixed with the fixed 1041 nm line (λ_2_), resulting in equivalent two-photon excitation at 760, 879, and 1041 nm. Four-channel multiphoton images were recorded, where epi-detected fluorescence was directed to three separate detectors and SHG produced by the 1041 nm beam was detected in transmission on a fourth detector. Fluorescence filters were chosen for rejecting coherent signals (SHG, SFG, FWM). As shown in Fig. 4a and b, blue fluorescence was efficiently excited at λ_1_, red fluorescence was efficiently excited at λ_2_, and green fluorescence was efficiently excited through two-color excitation when the pulse trains were temporally overlapped in the microscope (Fig. 4 and S6).

Zebrafish embryos (n = 9) were first imaged every 2 minutes during early development, from 4 cells to higher stages^40^ (Fig. 4c and video 1). Spectral imaging evidences major compositional differences between the dividing cells and the yolk. Cellular fluorescence is blue/green and can be attributed mostly to NADH and FAD, while the yolk exhibits strong green/red fluorescence possibly due to retinoids and oxidized lipids (Fig. 4c and d).

During divisions, the blue fluorescence pattern in cells reveals the spatial distribution of mitochondria, while SHG signal from mitotic spindles peaks at the end of metaphase^41^ (Fig. 4d). Spectral fluorescence imaging also revealed interesting changes over time in the yolk. First, an overall decrease of the red fluorescence was observed during the first hours of embryo development (Fig. 4c and video 1 in S1); we verified that this effect was not due to photo-bleaching (see Figure S7). In addition, fluorescence time-lapse imaging highlighted the shapes and dynamic behavior of yolk platelets (video 1)^41–43^. We found that adjacent yolk compartments have different spectroscopic characteristics (Fig. 4c), revealing different relative concentrations in fluorescent species. Moreover, the ratio between green and red fluorescence was found altering during the division stages and building up a gradient along the animal-vegetal yolk: yolk platelets that are closer to yolk syncytial layer (YSL, forming the interface with the dividing cells) exhibit stronger red fluorescence with respect to the yolk domains located further from the YSL (video 1 and video 2 in S1).

These observations suggest that spectral imaging of the embryo endogenous fluorescence provides a direct visualization of the mobilization of yolk nutrients and of metabolic gradients during cell cleavage and early development stages.

**Figure 5:**
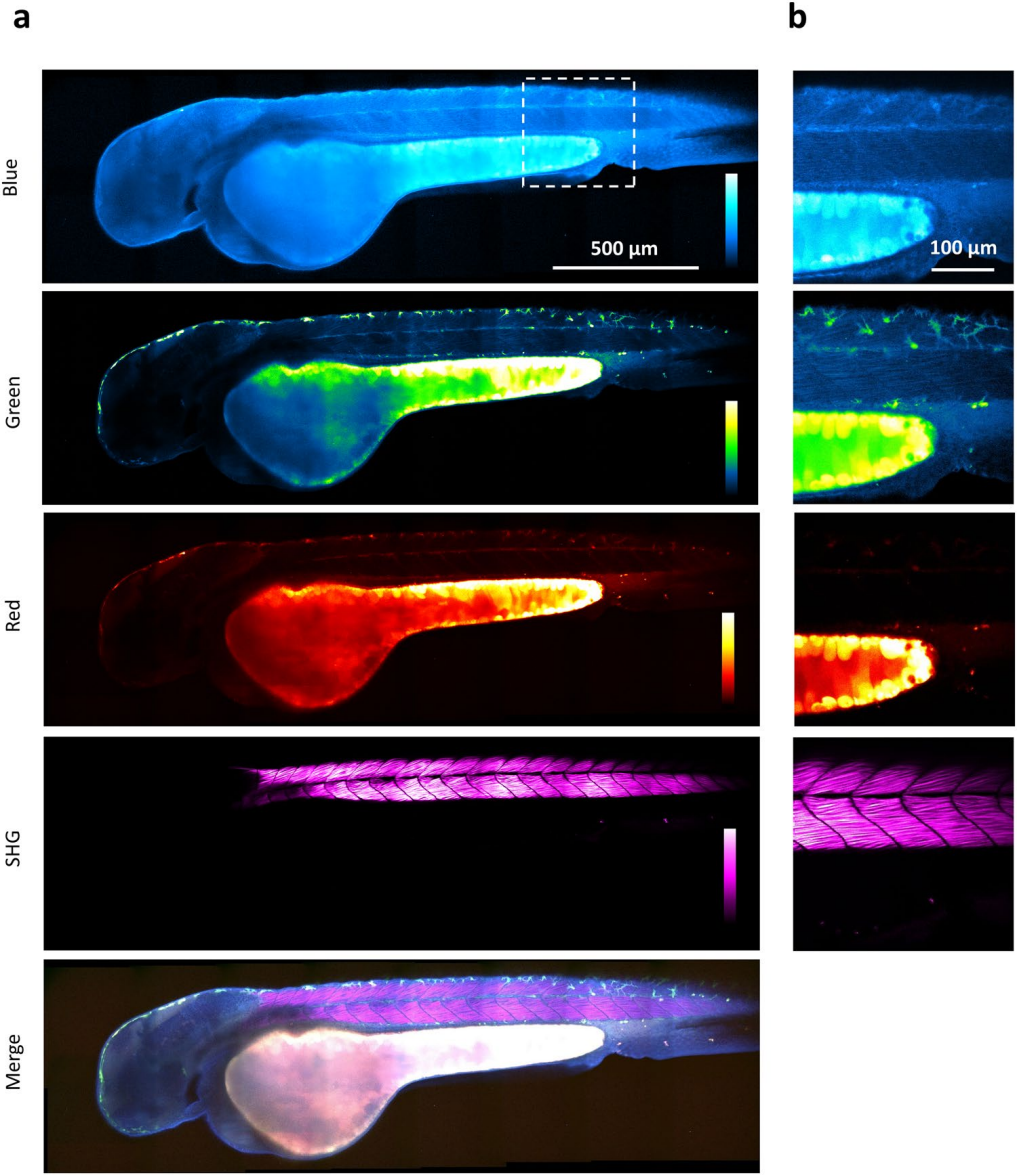
Multicolor two-photon efficient imaging of endogenous fluorophores in zebrafish embryo Images of the blue, green, red fluorescence, second harmonic generation, and merged channels of a live zebrafish embryo recorded 48 hours post fertilization (**a**) Large-scale (stitched mosaic) image encompassing 2450 × 730 µm^2^. (**b**) Detail extracted from the large-scale image. Pixel size, 0.83 µm. Multicolor pixel acquisition time, 40 µs. Scale bar, 500 µm.

We also recorded multicolor multiphoton images of endogenous signals at later stages of development. Figure 5 and video 3 show a zebrafish embryo at 48 hours post fertilization imaged using simultaneous trichromatic 2PEF and SHG signals. Here again, spectral multiphoton fluorescence imaging reveals the biochemical heterogeneity of yolk compartments. In addition, blue endogenous fluorescence, presumably dominated by NADH signals, provides detailed structural and possibly metabolic information from other regions such as the head and the trunk muscles. Strong SHG signals are obtained from myofilaments in the trunk, as reported in previous studies^3, 44^. Pigmented skin cells are particularly visible in the green channel.

## Conclusions

The present work implemented and demonstrated an efficient method for simultaneously imaging several endogenous fluorophores with different absorption spectra within a single measurement, which delivers intrinsic sub-micron registration of the color channels and is compatible with coherent nonlinear contrasts. Using mixed-wavelength two-photon excitation^33^, we demonstrated simultaneous excitation of endogenous blue, green and red intrinsic fluorophores along with SHG imaging in living tissues.

In particular, a simultaneous efficient two-photon excitation of NADH and FAD (Figs 1e, 2 and 3) was achieved in cells and tissues. In two-photon studies, NADH and FAD are usually excited sequentially at ~740 nm and ~900 nm. Their large difference in concentration^5^ limits simultaneous excitation of both fluorophores with a single wavelength, even if they have overlapping two-photon absorption spectra^2, 32^ (Fig. S1c and d). This work optimized NADH and FAD excitation efficiencies by controlling FAD signal level independently of NADH through two-color two-photon excitation (Fig. 1a and e), resulting in comparable fluorescence intensities in the two channels in a one-shot measurement. This method therefore permits fast intensity-based measurements of redox ratios to be performed without motion artifacts (Figs 2c, 4c and S5c) in dynamic systems such as developing embryos and altering tissues.

The simultaneous two-photon FLIM of NADH and FAD with comparable signal-to-noise ratios, resulting in comparable precision of the fluorescence lifetimes in both channels, was also demonstrated (Figs 2 and 3, S2, S3 and S5). This approach towards simultaneous measurement of NADH and FAD redox and FLIM gradients was applied and revealed changes in oxidative phosphorylation and glycolysis metabolic rates in living tissues, namely reconstructed human skin (Fig. 2) and adult *C. Elegans* germ line (Fig. 3). Two-photon FLIM is being increasingly used to study metabolic activity gradients such as the ones present across the different skin cell layers from epidermis to dermis layer in order to characterize skin pathologies^12, 23, 45^, and to monitor cell differentiation in living systems^9, 12, 17^. However, tissue imaging experiments were so far hampered by the necessary sequential excitation of NADH and FAD with a tunable laser.

Multicolor two-photon imaging of endogenous fluorophores by wavelength mixing therefore opens new perspectives for *in vivo* measurements in the context of biomedical applications and for non-invasive longitudinal studies in developmental biology.

Measuring redox ratio in a one shot reveals heterogeneity among cells and even within cells (Fig. 2c and S5c). The ability to simultaneously monitor the changes in redox ratio and in the NAD^+^/NADH and FAD/FADH_2_ ratio may give access to crucial information about the spatiotemporal dynamics of metabolic activity changes in tissues. Indeed, recent studies indicated that NADH and FAD fluorescence lifetimes and intensity redox ratio provide complementary information about the phenotypic heterogeneity in cancer tissue and can identify different subtypes of cells^12, 46, 47^. For the sake of caution, we note however that redox ratio measurements in thick tissue may be complicated by wavelength-dependent scattering. In contrast, FLIM measurements are less affected by variations in excitation intensity and fluorophore concentration.

Finally, the potential of our method was demonstrated to simultaneously excite multiple endogenous fluorophores in the 700–1000 nm range in developing zebrafish embryos (Figs 4 and 5), in combination with SHG imaging. Longitudinal measurements during embryo development revealed morphological and functional changes, signed by changes in the spectroscopic properties of endogenous fluorescence (Fig. 4c and video 1 and video 2). Within the yolk, a compositional heterogeneity between yolk compartments was found together with a global decrease in the red-to-green fluorescence ratio over time and the formation of a compositional gradient along the vegetal-animal axis (Fig. 4c).

This method allows changes in cellular redox ratio during early development to being characterized in a label-free manner. Of note, energy metabolic pathways play a crucial role in embryonic development and morphogenesis, establishing a complex interplay between epigenetic and tissue organization^48, 49^. Metabolic shifts in fact not only affect the energetic production of single cells, but also the activity of redox-sensitive transcription factors, which alter gene expression patterns^48^. Overall, the approach presented here can be used to track rapid metabolic changes during embryo development and provides a promising label-free optical tool for studying metabolic spatial-temporal changes *in vivo*.

## Methods

### Multicolor two-photon imaging

Imaging was performed on a lab-built laser scanning microscope. We used a dual-output infrared femtosecond laser source (Insight DS+, Spectra-Physics, Santa Clara, CA, USA) with a first beam tunable from 680 nm to 1300 nm (120 fs pulses, 80 MHz) and a second, fixed wavelength beam at 1041 nm (200 fs pulses). A dichroic mirror (Semrock, Rochester, NY, USA) was used to combine the two beams. Power in the two beams was controlled with motorized wave plates and polarizers. For wavelength mixing, beams were spatially overlapped at the sample plane using independent telescopes and mirrors^33^, and pulses were temporally overlapped using a delay line. Calibration of the delay line was performed by minimizing the delay between the two pulse trains and maximizing the sum frequency generation (SFG) signal from a KDP particle. For simultaneous excitation of blue, green and red endogenous fluorescence, excitation wavelengths λ_1_, λ_2_ and two-color equivalent excitation λ_v_ = 2/(1/λ_1_ + 1/λ_2_) wavelengths were set to 760 nm, 1041 nm and 879 nm, respectively. A water immersion objective (25×, 1.05NA, XLPLN25XWMP, Olympus, Tokyo, Japan) was used to focus the beam in the sample. Typical power was 10 mW for the 760 nm line and 40 mW for 1041 nm line. Signals were detected in the epi- and trans-directions by GaAsP modules (H7422P-40, Hamamatsu, Japan) or photomultiplier modules (SensTech, Langley, UK) and lab-designed counting electronics enabling time-correlated single photon counting for FLIM imaging (see below). Scanning and acquisition were synchronized using lab-written LabVIEW software and a multichannel I/O board (PCI-6115, National Instruments, Austin, TX, USA). Fluorescence was collected in the backward (epi) direction using a dichroic mirror (695dcxru, Chroma, USA) and 2PEF signals were directed toward three independent detectors using dichroic mirrors (Semrock 520 nm and 562 nm). Bandpass filters were used in front of the detectors to collect blue (Semrock FF01–466/40), green (Semrock FF01–550/49) and red (Semrock FF01–650/40) fluorescence, respectively. SHG was collected in transmission with a bandpass filter 520/15 nm (Semrock FF01–520/15). Scanning was performed by galvanometric mirrors (VM500S, GSI Lumonics, Bedford, MA, USA). Typically the acquisition time of intensity images was on the order of few seconds, with a pixel dwell time of 20 μs.

### Fluorescence Lifetime Imaging Microscopy

FLIM data are acquired simultaneously in two channels with a custom made TCSPC electronic card programmed on FPGA with 24 temporal bins of 500 ps. For every laser pulse, the electronics counts single photons in a histogram according to their arrival time. The histogram has 24 temporal bins, each bin width is 1/(24*ν) = 0.52 ns, where ν = 80 MHz is the laser frequency. The arrival time between the photon and the laser pulse is measured by a Time to Digital Converter (TDC). The laser pulse time, detected by a photodiode (Thorlabs PDA10CF-EC) is the start of the TDC. The photons arrival time from the PMT (Hamamatsu H7422P-40) is the stop of the TDC. The pulses of the PMT are detected by a fast discriminator (Hamamatsu C9744 photon counting unit). The random amplification mechanism and the transit time spread of the detector induce a timing jitter. Since we measure a jitter after the discriminator of 400 ps, which is smaller than the temporal window of 500 ps, we do not add a Constant Fraction Discriminator (CFD). Calibration of the system is performed by measuring the lifetime of the SHG (0 ns) and lifetime of fluorescein at pH9 with a single exponential of 4.04 ns (see Fig. S2a). Typically, the acquisition time of FLIM images is on the order of 4 minutes for a 320 × 320 pixels image at a pixel dwell time of 160 μs/pixel (eight images with 20 μs/pixel are accumulated).

### Data analysis and processing

All intensity data were analyzed and treated with ImageJ (NIH, Bethesda, MD, USA). All FLIM data were processed and analyzed with a Matlab (Mathworks, Natick, MA, USA) custom written software. Every pixel of the FLIM image was transformed in one pixel in the phasor plot as previously described^9, 35^ and reported in detail in the Supplementary information. The coordinates *g* and *s* in the phasor plot were calculated from the fluorescence intensity decay of each pixel of the image (Fig. 1d) by using the transformations defined in the Supplementary material. We applied a median filter on the *g* and *s* images to reduce the variance of the phasor location without decreasing the spatial resolution of the image^50^. For every pixel of the image, we calculated the value of *τ*_*φ*_ (equation 5 in Supplementary material) starting from the g and s images. We represented the FLIM data with the *τ*_*φ*_ map.

### Reconstructed human skin

Reconstructed human skin samples (T-Skin™ model) were purchased from Episkin™ (Lyon, France) at day 11. T-Skin™ is a full thickness skin model containing both living dermis and epidermis covered by a well-structured *stratum corneum*. The dermal layer contains normal human fibroblasts embedded in a collagenous substrate supporting successive keratinocyte layers: the basal layer, located right above the dermal substrate, contains proliferating undifferentiated cells, while the uppermost layer, the *stratum corneum*, contains fully differentiated keratinocytes, i.e. corneocytes. This model is histologically similar to the *in vivo* human skin. The T-Skin™ samples, reconstructed at day 11, were placed between two glass coverslips with a few µl of culture medium (Episkin Assay Medium, Episkin™, Lyon, France) and imaged immediately after preparation.

### *C. Elegans* Germ line

The *C. Elegans* strain used in this study was the Bristol wild-type N2 variety. Nematodes were grown at 20 °C on NGM plates seeded with E. coli strain OP50. To mount the nematodes, a 10% agarose pad was extemporaneously prepared on a coverslip. Young adults or L4 larvae were transferred in a drop of 0.1 µm polystyrene beads (Polyscience Inc. cat 00876, Warrington, PA, USA) without any anesthetic drug. A coverslip was added and animals were immediately imaged.

### Zebrafish embryo

Embryos were obtained from a wild type fish line (TU) and provided by Amagen facility at Paris Saclay University. Embryos were imaged at 3 and 48 hpf. Imaging of zebrafish early embryonic development was performed by embedding the embryos in 1% Low Melting Point agarose (Invitrogen, cat 15517*–014*, Carlsbad, CA, USA*)*. Embryos were kept inside their chorions. Imaging of 48hpf embryos was performed by embedding them in 1.5–2% Low Melting Point agarose and adding tricaine (MS-222 Sigma, cat E10521, St. Louis, MO, USA) to a final concentration of 0.02%.

### Solution preparation

A solution of 250 μM NADH (Sigma Aldrich n. N8129, St. Louis, MO, USA) was prepared in 100 mM Mops buffer at pH 7. FAD solution (Sigma Aldrich n. F6625, St. Louis, MO, USA) was diluted at 2 mg/ml in water.

### NIH 3T3 mouse embryonic fibroblast cells

NIH 3T3 cells were purchased from the American Type Culture Collection (ATCC, Manassas, VA, USA). NIH3T3 mouse fibroblast cells were cultured on 3.5-cm glass bottom petri dishes (MatTek, Ashland, MA, USA) in Dulbecco’s Modified Eagle’s Medium (DMEM) modified without phenol red and containing 10% FBS and 1% penicillin/streptomycin. Cells were plated at the initial density of 5,000 cells/cm^2^ and allowed to attach overnight in a humidified cell culture incubator at 37 °C in 5% CO_2_/95% air before proceeding with treatments.

## Electronic supplementary material

Supplementary Info and Figures

Video 1

Video 2

Video 3
