# Supplementary material for: Multicolor two-photon imaging of endogenous fluorophores in living tissues by wavelength mixing
Source: Sci Rep. 2017 Jun 19;7:3792. doi: 10.1038/s41598-017-03359-8 (PMC5476668; doi:10.1038/s41598-017-03359-8)
Supplement: Supplementary file 1 — Supplementary Info and Figures [file 41598_2017_3359_MOESM1_ESM.pdf]

Supplementary Information and Figures

Multicolor two-photon imaging of endogenous fluorophores in living tissues by wavelength mixing

Chiara Stringari[1], Lamiae Abdeladim[1], Guy Malkinson[1], Pierre Mahou[1], Xavier Solinas[1], Isabelle Lamarre[1], Sébastien Brizion[2], Jean-Baptiste Galey[2], Willy Supatto[1], Renaud Legouis[3], Ana-Maria Pena[2], Emmanuel Beaurepaire[1]

1) Laboratory for Optics and Biosciences, Ecole polytechnique, CNRS, INSERM, Université Paris-Saclay, 91128 Palaiseau cedex, France.

2) L'Oréal Research and Innovation, 93600 Aulnay sous Bois, France

3) Institute for Integrative Biology of the Cell (I2BC), CEA, CNRS, Univ. Paris-Sud, Université Paris-Saclay, 91198 Gif-sur-Yvette, France.

SUPPLEMENTARY INFORMATION:

The phasor analysis of fluorescence lifetime data

The fluorescence lifetime intensity decays in every pixel of the image are transformed in the phasor plot through a Fourier transform. The components *g* (x-coordinate) and *s* (y-coordinate) are given by the following expressions:

$$g_{i,j}(\omega) = \frac{\int_0^\infty I_{i,j}(t)\cos(\omega t)dt}{\int_0^\infty I_{i,j}(t)dt} \qquad (1)$$

$$s_{i,j}(\omega) = \frac{\int_0^\infty I_{i,j}(t)\sin(\omega t)dt}{\int_0^\infty I_{i,j}(t)dt} \qquad (2)$$

where the indices *i* and *j* identify a pixel of the image and ω the frequency (ω=2πf), where *f* is the laser repetition rate, *i.e.* 80 MHz in our experiment. All phasor plots are calculated at 80 MHz, *i.e.* the first harmonic of the laser repetition rate.

The phasor transformations of FLIM data in the frequency domain are:

$$g_{i,j}(\omega) = m_{i,j}\cos\varphi_{i,j} \qquad (3)$$

$$s_{i,j}(\omega) = m_{i,j}\sin\varphi_{i,j} \qquad (4)$$

where $m_{i,j}$ and $\varphi_{i,j}$ are the modulation and the phase of the emission with respect to the excitation. Estimations of the lifetime in terms of the phase and modulation can be performed in each pixel by the following formulas:

$$\tau_\varphi = \frac{1}{\omega}tan(\varphi) \qquad (5)$$

$$\tau_m = \frac{1}{\omega}\sqrt{\left(\frac{1}{m^2} - 1\right)} \qquad (6)$$

In the case of a single exponential decay, the two lifetimes obtained by the phase and by the modulation with equations (5) and (6) are equal, while for a multi-exponential lifetime system the apparent lifetimes are different.

SUPPLEMENTARY FIGURES:

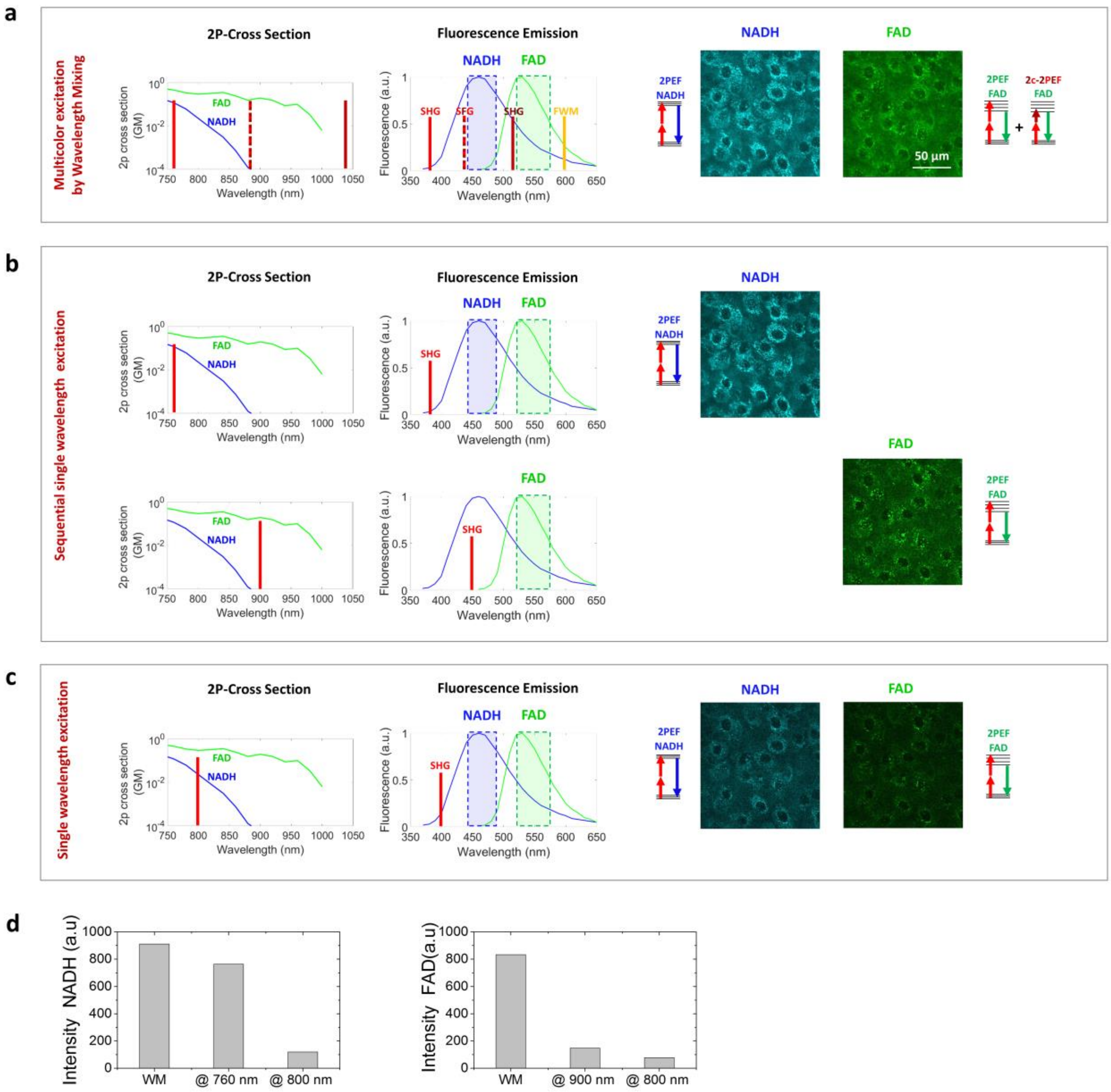

Figure S1. NADH and FAD excitation efficiency of wavength mixing method compared to sequential single wavelength excitation at 760nm and 900nm and single excitation at 800nm. (a) Wavength mixing allows simultaneous and efficient excitation of NADH and FAD. 10 mW for the 760 nm line and 40 mW for 1041 nm line were used. **(b)** Sequential single wavelength excitation at 760nm and 900nm allow efficient excitation of NADH and FAD, but requires sequential excitation with a tunable laser, i.e, not simultaneous acquisitions. 10 mW power was used for both the 760 nm and 900nm excitation wavelength. **(c)** Single excitation at 800nm permits simultaneous excitation of NADH and FAD (first proposed by Huang *et al. Biophysical Journal 2002),* by compensating the differences in absorption spectra and concentration. However this method is not efficient, hence it requires long exposure times, which are incompatible with dynamic living systems. 10 mW were used for the 800nm excitation. **(d)** Comparison of fluorescence intensity of NADH and FAD channels obtained with the three different methods described in (a-c).

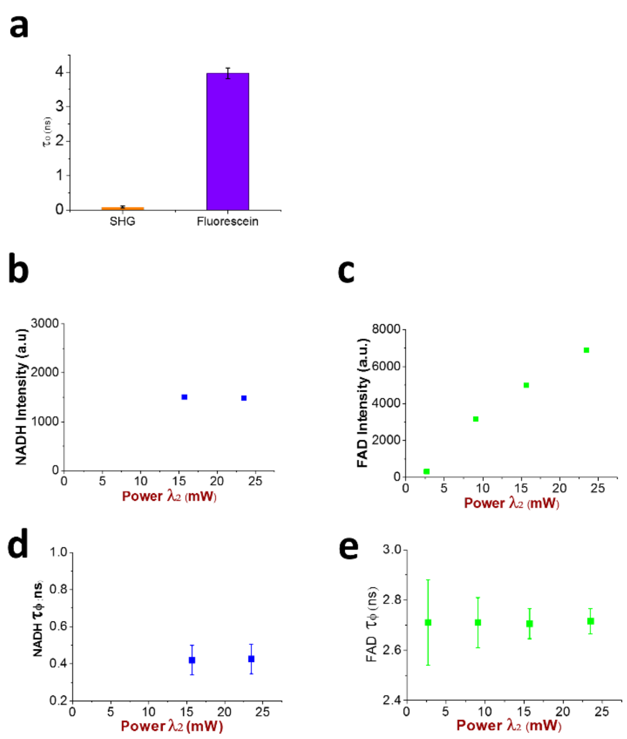

Figure S2. Fluorescence lifetime of NADH and FAD is not affected when the fluorophores are excited through wavelength mixing. (a) Lifetime calibration of the FLIM system is performed on a second harmonic signal from KDP particle and fluorescein solution at pH=9. **(b-e)** Dependence of the NADH and FAD intensities (b-c) and lifetime (d-e) on the power of the laser line λ_2=1040nm. Measurements were performed with fluorophores in solution.

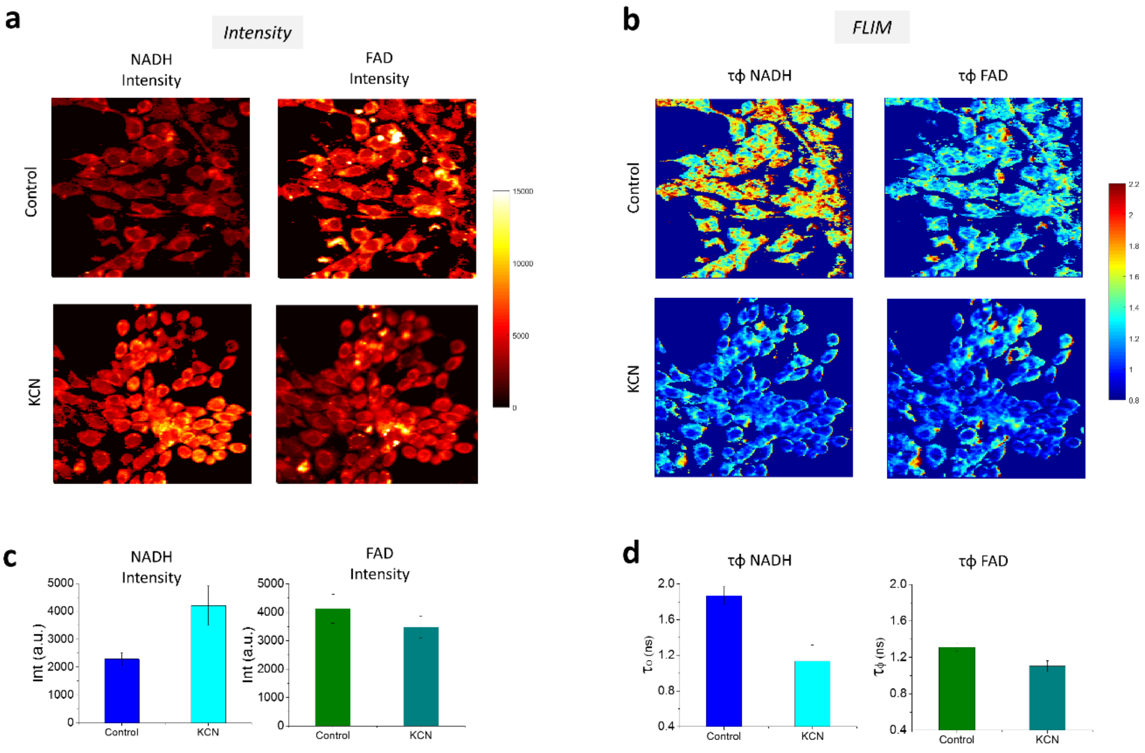

Figure S3. Intensity and fluorescence lifetime changes in NIH 3T3 mouse embryonic fibroblast cells upon application of oxidative phosphorylation blocker potassium cyanide (KCN). (a-b) NADH and FAD intensities (a) and fluorescence lifetimes (b) before and after application of KCN. **(c)** Upon KCN application NADH intensity increased while FAD intensity decreased. **(d)** Upon KCN application NADH and FAD lifetime decreased.

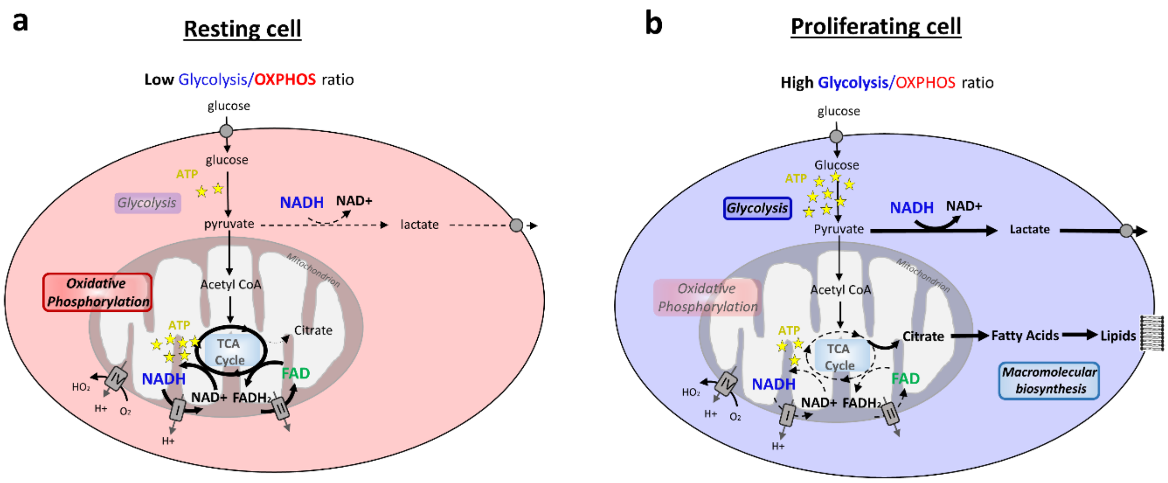

Figure S4. Schematic representation of the differences between the metabolism of a resting and a proliferating cell. (Derived from reference [38])

Glucose breakdown through glycolysis and the TCA cycle (dark blue) generates reduced NADH and $FADH_2$. **(a)** Quiescent cells have a basal rate of glycolysis, converting glucose to pyruvate, which is then oxidized in the TCA cycle. As a result, the majority of ATP (yellow stars) is generated by oxidative phosphorylation. Nonproliferating (differentiated) cells are characterized by a low NADH/NAD+ ratio and a high FAD/(NADH+FAD) ratio. **(b)** During proliferation, the large increase in glycolytic flux rapidly generates ATP in the cytoplasm. Most of the resulting pyruvate is converted into lactate by lactate dehydrogenase A, which regenerates NAD+ from NADH. Proliferating cells are characterized by a high NADH/NAD+ ratio and a low FAD/(NADH+FAD) ratio.

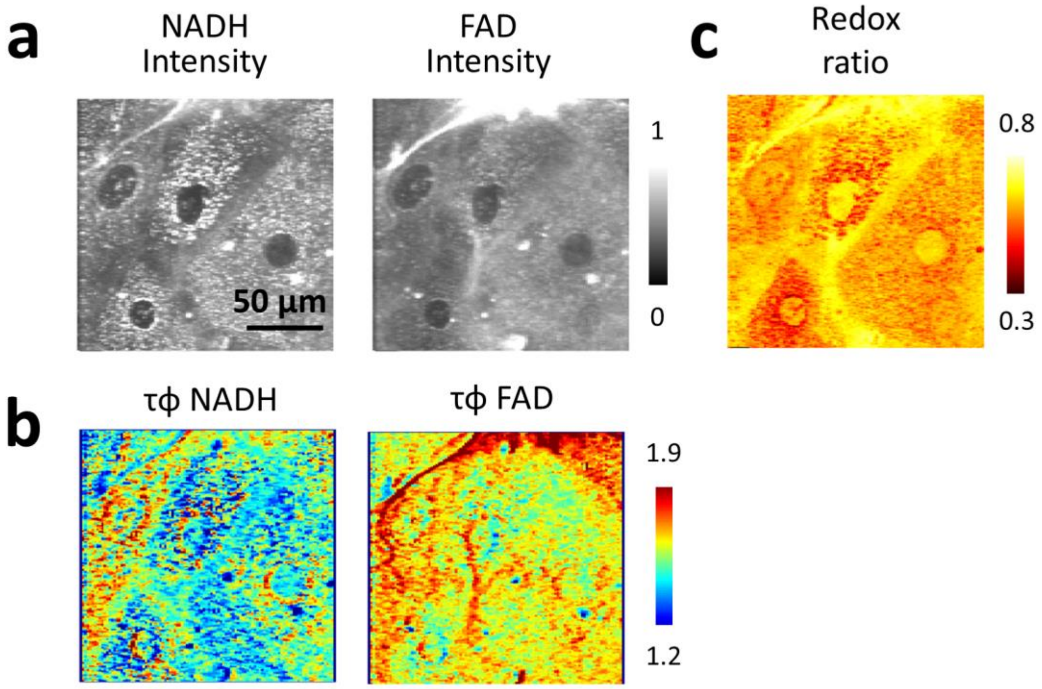

Figure S5. Simultaneous multiphoton imaging of NADH and FAD reveals heterogeneity in the intensity redox ratio and NADH and FAD lifetime within the tissue (a) Simultaneously acquired NADH and FAD intensities**(b)** NADH and FAD fluorescence lifetime maps **(c)** Intensity redox ratio FAD/(NADH+FAD), calculated from NADH and FAD intensities reveal heterogeneity of metabolism in the tissue on a pixel base. In some pixels of the image there is a correlation between the intensity redox ratio and the NADH fluorescence lifetime map.

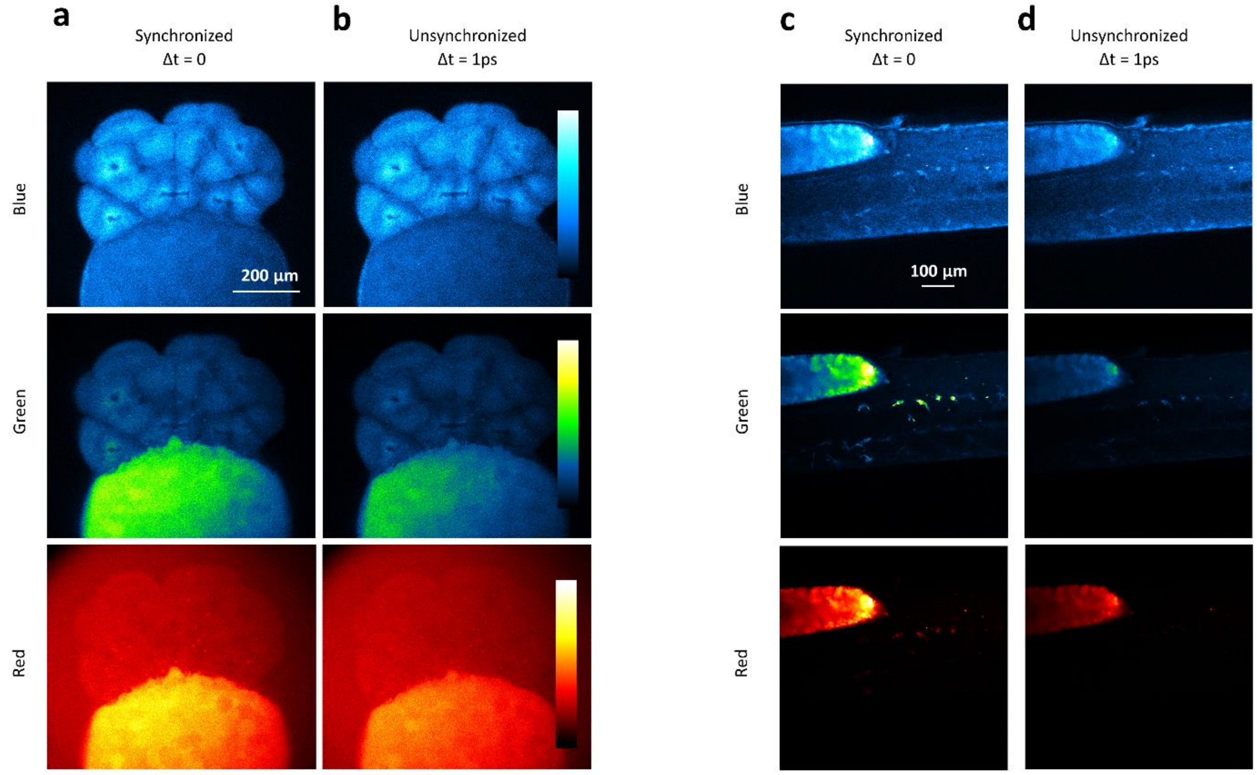

Figure S6. Enhancement of the green fluorescence by wavelength mixing in zebrafish embryo. Images of the blue, green, red, SHG and merged channels in zebrafish embryos at early stages of development **(a-b)** and at 48 hours developmental stage **(c-d).** Fluorescence images using synchronized (Δt=0) **(b-d)** and unsynchronized (Δt=1ps) pulse trains **(a-c)**.

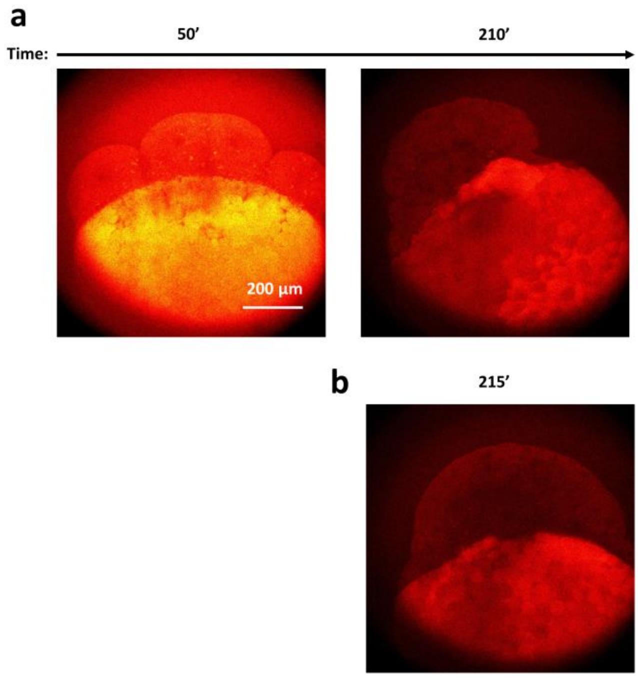

Figure S7 Decrease of the red autofluorescence is not due by photo-bleaching

Red fluorescence channel of two embryos imaged from 8 stage cell (a) and from high state (b)

Movies Caption

Movie M01: **Multicolor two-photon imaging and SHG imaging during early stages of zebrafish embryo development**
Time-lapse, multicolor two-photon imaging and SHG imaging from the four-cell stage can track cell division during embryo development. Images are acquired every 2 minutes. Images of merged blue, green, red channels and SHG in white. Raw data visualized with ImageJ software.

Movie M02: **Multicolor two-photon imaging during early stages of zebrafish embryo development**
Time-lapse, multicolor two-photon imaging from the sixteen-cell stage. Images are acquired every 2 minutes. Images of merged blue, green, red channels. Raw data visualized with ImageJ software.

Movie M03:
Multicolor two-photon efficient imaging and SHG imaging of endogenous fluorophores in zebrafish embryo.
Multicolor two-photon imaging of the zebrafish embryo at 48 hours development. Raw data visualized with Imaris software.
